# Disaggregating catastrophic health expenditure by disease area: cross-country estimates based on the World Health Surveys

**DOI:** 10.1186/s12916-019-1266-0

**Published:** 2019-02-13

**Authors:** Annie Haakenstad, Matthew Coates, Andrew Marx, Gene Bukhman, Stéphane Verguet

**Affiliations:** 1 Department of Global Health and Population, Harvard T.H. Chan School of Public Health, 665 Huntington Avenue, Boston, MA 02115 USA; 2 Department of Global Health and Social Medicine, Harvard Medical School, 641 Huntington Avenue, Boston, MA 02115 USA

**Keywords:** Catastrophic health expenditure, Out-of-pocket spending, Financial risk protection, Universal health coverage, Illness-related impoverishment, Poverty

## Abstract

**Background:**

Financial risk protection (FRP) is a key objective of national health systems and a core pillar of universal health coverage (UHC). Yet, little is known about the disease-specific distribution of catastrophic health expenditure (CHE) at the national level.

**Methods:**

Using the World Health Surveys (WHS) from 39 countries, we quantified CHE, or household health spending that surpasses 40% of capacity-to-pay by key disease areas. We restricted our analysis to households in which the respondent used health care in the last 30 days and categorized CHE into disease areas included as WHS response options: maternal and child health (MCH); high fever, severe diarrhea, or cough; heart disease; asthma; injury; surgery; and other. We compared disease-specific CHE estimates by income, pooled funding as a share of total health expenditure, share of the population affected by the different diseases, and poverty status.

**Results:**

Across countries, an average of 45.1% of CHE cases could not be tied to a specific cause; 37.6% (95% UI 35.4–39.9%) of CHE cases were associated with high fever, severe cough, or diarrhea; 3.9% (3.0–4.9%) with MCH; and 4.1% (3.3–4.9%) with heart disease. Injuries constituted 5.2% (4.2–6.4%) of CHE cases. The distribution of CHE varied substantially by national income. A 10% increase in heart disease prevalence was associated with a 1.9% (1.3–2.4%) increase in heart disease CHE, an association stronger than any other disease area.

**Conclusions:**

Our approach is comparable, comprehensive, and empirically based and highlights how financial risk protection may not be aligned with disease burden. Disease-specific CHE estimates can illuminate how health systems can target reform to best protect households from financial risk.

**Electronic supplementary material:**

The online version of this article (10.1186/s12916-019-1266-0) contains supplementary material, which is available to authorized users.

## Background

Universal health coverage (UHC) aims to ensure that all people have access to quality health services while also providing protection from health care-related financial hardship [1]. UHC is aspirational and multidimensional. As emphasized by the World Health Organization (WHO) and the World Bank and included in target 3.8 of the Sustainable Development Goals (SDGs), UHC has become a major global health priority [2].

Financial risk protection (FRP) is a core pillar of UHC and a major aim of health systems [3, 4]. In 2010, an estimated 210 million people incurred catastrophic health expenditure (CHE), a key measure of FRP, defined as out-of-pocket (OOP) health expenditure that surpasses 40% of non-food expenditure [5]. OOP health spending can push households into poverty and further impoverish households already below the poverty line; it can also act as a deterrent to accessing health services [6–8].

Noncommunicable diseases and injuries (NCDIs) are increasingly an important part of disease burden among the world’s poorest people but have been neglected by health financing, particularly by development assistance for health [9, 10]. NCDIs could be a significant cause of impoverishment and CHE in the developing world—cancers, heart disease, and major injuries can entail major treatment costs [11]. Understanding the distribution of CHE by disease area, including NCDIs, is thus critical to pinpointing how national health systems may fall short in protecting households from the financial risks of disease and supporting countries in setting priorities to enhance FRP in pursuit of UHC.

However, only a few studies have assessed the incidence of CHE associated with NCDIs, and to our knowledge, no research to date has systematically contrasted CHE driven by NCDIs to CHE driven by other disease areas across countries in a comparable and comprehensive manner. While many studies of disease-specific national health expenditure have been spurred by the System of National Health Accounts 2011 (SHA 2011), only a limited number of national health accounts break down OOP spending by disease area or quantify OOP spending related to NCDIs. Of 100 disease-specific national health accounts made available by the WHO, only 14 capture OOP for NCDs [12, 13]. Recently, Essue and colleagues summarized a variety of studies estimating CHE for select NCDs, but assumed the same utilization and cost patterns applied to disparate country contexts to generate global CHE estimates [14]. Jan et al. conducted a systematic review of existing NCD CHE literature and were unable to standardize CHE across studies, limiting the number of cross-country comparison that could be made [15], while Verguet et al. developed a mathematical framework to assess medical impoverishment by cause and applied it to a systematic categorization by disease in Ethiopia [16]. Often, existing studies on FRP and OOP capture health expenditure and CHE for one specific disease at a time, precluding comparison across diseases. Furthermore, many analyses are characterized by methodological idiosyncrasies, such as convenience sampling or episode-based spending, or have a limited disease focus that make comparison across studies and countries difficult [11, 17].

We address this gap in knowledge by using the World Health Surveys (WHS) to characterize the distribution of CHE across key disease areas in 39 low- and middle-income countries [5]. We report CHE by disease according to World Bank income group, the share of total health expenditure that is pooled, the share of the population affected by specific diseases, and poverty status.

## Methods

We used the WHS as our primary source of data. The WHS was implemented over 2002–2004 in 39 low- and middle-income countries (listed in Additional file 1) and surveyed more than 238,000 respondents. It deployed a multistage sampling design to capture a nationally representative population. The WHS was selected because it is the only survey implemented to date that captures the reason for seeking care and associates care-seeking with spending in more than six developing countries. Thus, the WHS serves as one of the only existing data sources that can be used to empirically compare CHE by disease across countries.

The WHS collected household expenditure for a range of items. We focused our analysis on health, food, and total consumption expenditure. First, households were asked to report total expenditure over the last 4 weeks, which we used as our measure of household financial well-being. Second, households reported monthly spending on food: we used the mean of the 45th–55th percentiles of these expenditures as our subsistence expenditure threshold, adjusted for household size, consistent with previous CHE studies [5]. Third, to calculate monthly health spending, we summed households’ 30-day expenditure on inpatient care, outpatient care, care from traditional providers, medicines, diagnostics, and other health care costs.

Subsequently, we paired total household expenditure with spending on food and health to calculate CHE. We defined capacity-to-pay as the difference between total household expenditure and the subsistence expenditure threshold, calculated as the mean of 45th–55th percentile of food expenditure [18, 19]. This was adjusted for the number of household members by using an exponent of 1/2 to scale the subsistence expenditure threshold, following the established literature [19]. For households spending less than the subsistence threshold, we represented capacity-to-pay with reported non-food spending. Thirty-day health expenditure was deemed catastrophic if it comprised more than 40% of capacity-to-pay. We chose this measure of CHE because it is the most commonly used in the literature and is more sensitive to CHE among the poor [5].

We then used unique WHS questions about the causes of health care utilization to identify the diseases and conditions associated with health expenditure. The WHS asked the respondents detailed questions about their most recent health care encounter. Specifically, the respondents were asked: “Which reason best describes why you [your child] last needed health care?” The respondents could select among 12 response options. We grouped four of these options into a maternal and child health (MCH) category: “antenatal consultation,” “family planning,” “immunizations,” and “child birth.” We also created a maternal care only category (excluding immunizations). We reported separately each of the following response options: “high fever, severe diarrhea, or cough,” “heart disease,” “asthma,” “injury,” and “minor surgery.” We grouped “other” and “arthritis” into the “other” category because we assumed arthritis was interpreted by respondents as pain in joints or other generalized pain that would unlikely truly be arthritis. We omitted the response option “dental care.”

We restricted our analysis to households in which the respondent used health care in the last 30 days to match utilization with spending: households were asked to report total health expenditure over the same time period (30 days). We categorized CHE according to the cause of utilization selected by the respondent.

We integrated the complex survey design of the WHS to bootstrap the uncertainty of CHE by disease. We designated the different strata used to select clusters and resampled at the strata level, allowing us to maintain the national representativeness of the survey while also integrating the unique survey design implemented in each country. We took *n* = 1000 draws of the underlying data to calculate bootstrapped uncertainty intervals (UIs) (5th and 95th percentiles, respectively) for estimating CHE incidence. Finally, to scale up to a global level, we used the population of each country at the time of the WHS to weight estimates.

We also calculated a modified version of the multidimensional poverty index (MPI) [20], which we called the poverty index (PI). The MPI measures poverty according to the number of deprivations a household experiences in health, education, and living standards. Because our focus was health, we omitted the health deprivation in the PI, avoiding concerns about confounding. The WHS did not capture whether household members of the appropriate age were attending primary school and thus the education portion of the PI was based on whether adults in the household had completed primary school. Additional file 1: Table S1 lists each indicator and its definition. Under the PI, households were considered poor when deprived in four or more areas. Using this classification, we compared CHE by disease between poor and non-poor households.

To understand the association between disease-specific CHE and the share of the population affected by the specific diseases and conditions captured in the WHS, we used 2016 Global Burden of Disease (GBD 2016) estimates for the incidence of injuries and the prevalence of cardiovascular disease (CVD) for the years 2002–2004 [21]. Because spending on maternal care captured all deliveries, we compared maternal care CHE with the crude birth rate as a share of the population, as estimated by the United Nations Population Division [22]. These cross-sectional comparisons highlight how the share of the population affected by CHE differed by disease area and how well health systems adapted their financial risk protection measures to the most prominent areas of disease nationally. A positive relationship would suggest that as the share of the population afflicted rises, more people would incur CHE. Comparing these relationships across disease areas indicates how well countries protect people from one area of disease versus another, and thus responds to the financial risks associated with the distribution of disease in their population.

We depict the distribution of CHE by disease according to two defining features of health systems: income and the pooling of health financing. First, we define income using the World Bank income groups. Income is highly correlated with how much a country can spend on health and thus invest in financial risk protection [23]. Income is also correlated with the share of the population living below the poverty line and thus most susceptible to CHE. Second, we use the share of health expenditure that is pooled (sourced from governments and prepaid private contributions) as estimated by the World Health Organization [24]. Pooled financing represents how well the health system is organized to protect people financially from health care costs and how much is invested in financial risk protection at the national level.

Finally, we examined the relationship between the share of the population affected by each of the highlighted disease areas and key health system features. We regressed each of the disease-specific CHE measures on the respective measures of the share of the population affected and, in multivariate regressions, controlled for gross domestic product (GDP) per capita, pooled funding as a share of the total health expenditure (Pooled/THE), and government health expenditure as a source (GHES) per capita, in order to assess whether the relationship between the share of the population afflicted by the disease and diseases-specific CHE, respectively, could be explained by underlying features of the health system, including its organization and financing [25]. Finally, we assessed the association between the “other” CHE category and measures of the prevalence of NCDs and communicable causes not otherwise captured in the WHS, standardized by subtracting mean prevalence and dividing by the standard deviation across countries. Additional file 1 presents the causes included and how prevalence estimates were computed.

All analyses were conducted in Stata 14.0.

## Results

Table 1 depicts select indicators for each World Bank income group. On average, 5603 households were surveyed in each country, ranging from 1028 in Bosnia and Herzegovina to 38,746 in Mexico. The share of households incurring CHE ranged from 30% in lower-middle-income countries to 15% in low-income countries. These estimates are similar to other CHE estimates using the WHS, but slightly higher than estimates that do not use these surveys [5, 26].

**Table 1:** Summary indicators, across the different country income groups included in the analysis

Income group (*N*)	Average number of respondents	Mean age (years)	Average share of female respondents	Average share rural	Average share of population with an outpatient visit in the last year	Share with catastrophic health expenditure
LICs (8)	4596	38	54%	77%	29%	15%
LMICs (18)	4906	40	51%	81%	58%	30%
UMICs (13)	7124	45	54%	53%	46%	17%

Notes: *LICs* low-income countries, *LMICS* lower-middle-income countries, *UMICs* upper-middle-income countries, according to 2002–2004 World Bank income classifications. Survey weights used at the national level; population size used to weight at the income level. No population weights used for average number of respondents. Poverty index defined according to the number of deprivations in education and assets, a modified version of the multidimensional poverty index

Across all 39 countries, 37.6% (95% UI 35.4–39.9%) of CHE cases were associated with fever, cough, or diarrhea. The largest category was “other”: we were unable to associate 45.1% (42.6–47.6%) of CHE cases with a specific disease area. MCH and heart disease were associated with 3.9% (3.0–4.9%) and 4.1% (3.3–4.9%) of CHE cases, respectively. Injuries constituted a slightly higher share of all CHE cases, at 5.2% (4.2–6.4%), while asthma CHE was slightly lower, at 3.0% (2.2–3.9%). Although “minor surgery” was associated with less than 2% of cases, some surgical spending would likely be associated with the other categories and thus the “minor surgery” estimates should be interpreted as a lower bound of CHE associated with surgical care. Much more CHE variation was observed with respect to heart disease than any other area. The standard deviation for heart disease CHE was 4.5 per 1000, larger than the standard deviations of maternal-, injury-, and asthma-associated CHE, at 1.9, 1.8, and 1.4 per 1000, respectively.

**Fig. 1:**
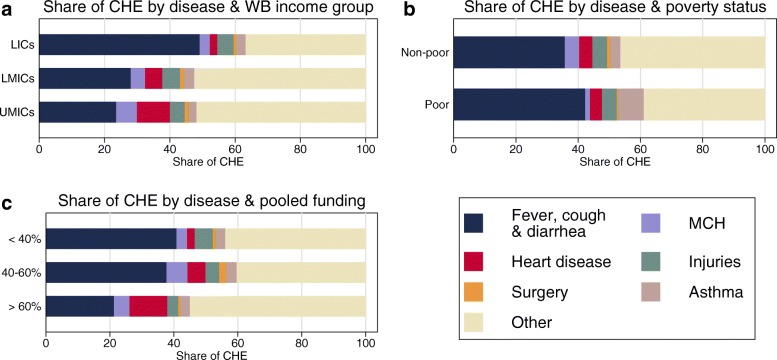
Share of catastrophic health expenditure (CHE) by disease grouped by the World Bank income group, poverty status, and pooled funding. Among households with a respondent that used health care in the last 30 days. LICs: low-income countries; LMICS: lower-middle-income countries; UMICs: upper-middle-income countries; according to 2002–2004 World Bank income classifications (**a**). Households considered poor according to a modified version of the multidimensional poverty index (**b**). Pooled funding: share of prepaid private and government spending as a share of total health expenditure, countries grouped by the interquartile range of: less than the 25th percentile (< 40% pooled), 25th–75th percentile (40–60% pooled), and more than the 75th percentile (> 60% pooled) (**c**). Survey weights used at the national level; population size used to weight across countries

We also report on the distribution of CHE by disease area disaggregated by three groupings (Fig. 1a). First, we depict the distribution of CHE by disease across World Bank income groups. Distinct patterns in the distribution of CHE by income group emerged: as income rises, a smaller share of CHE was related to fever, diarrhea, and cough, and a larger share of CHE was associated with heart disease and the other category. The portion of CHE associated with injuries and MCH were generally stable across income levels. Second, we represent the distribution of CHE by disease across pooled financing as a share of THE, grouped by the 25th percentile (< 40% pooled), the 25th–75th percentile (40–60% pooled), and the 75th percentile (> 60% pooled). In countries with the greatest pooling, a larger share of CHE was associated with heart disease and the other category. Third, we compare the pattern of CHE between poor and non-poor households (Fig. 1b). The fraction of CHE due to fever, diarrhea, and cough was somewhat higher among the poor (42.3%, 95% UI 34.6–50.4%) as compared to the non-poor (35.8%, 33.1–38.8%). Asthma CHE was also slightly higher among the poor (8.3%, 3.0-14.8%) than the non-poor (3.3, 2.2-4.4%). However, the distribution of CHE cases by PI status was not distinct for all other causes. Additional file 1: Tables S2-S3 provides estimates for the distribution of CHE overall and by income and poverty status.

**Fig. 2:**
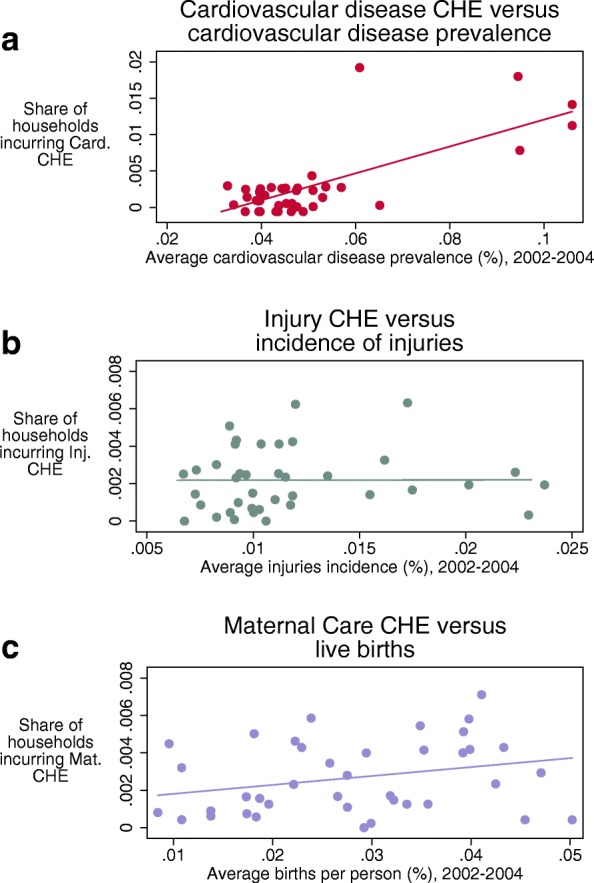
Comparing catastrophic health expenditure (CHE) to the share of the population affected by disease area (cardiovascular disease (**a**), injuries (**b**), and maternal care (**c**)). Among households with a respondent that used health care in the last 30 days. CHE: catastrophic health expenditure defined as 40% of capacity-to-pay. Source of prevalence of cardiovascular disease and incidence of injuries from Global Burden of Disease Study 2016. Source of live births from United Nations Population Division

Figure 2 captures the association between disease-specific CHE and the share of the population affected by each disease, using different metrics based on the disease or condition profile. The slope of the association of CVD prevalence and heart disease CHE was the highest. The slope of the association between maternal care CHE and live births per person was less pronounced, and the slope for the relationship between injuries and CHE was effectively flat. The visualization was confirmed by our univariate and multivariate regressions (Table 2): the coefficient for CVD prevalence was positive (0.10, 95% UI 0.13–0.24)—a result that is robust to controlling for GDP per capita, GHES per capita, and the share of health expenditure that is pooled. The coefficients for live births per person (0.05, 95% UI − 0.01–0.10) and injury incidence (0.00, UI − 0.14–0.14) were smaller and not statistically significant in the univariate regressions. In the multivariate regression including pooled/THE and GHES per capita, the coefficient on live births per person was statistically significant (0.13, UI 0.03–0.23).

**Table 2:** Results from regressing catastrophic health expenditure (CHE) on the share of the population affected, by disease area

	(1) Heart disease CHE	(2) Heart disease CHE	(3) Heart disease CHE	(4) Injuries CHE	(5) Injuries CHE	(6) Injuries CHE	(7) Maternal CHE	(8) Maternal CHE	(9) Maternal CHE
Cardiovascular disease prevalence	0.19***	0.17***	0.15***						
	[0.13 to 0.24]	[0.11 to 0.22]	[0.08 to 0.22]						
Injuries incidence				0.00	0.02	0.02			
				[− 0.14 to 0.14]	[− 0.13 to 0.16]	[− 0.20 to 0.24]			
Crude birth rate (percent)							0.05	0.05	0.13**
							[− 0.01 to 0.10]	[− 0.03 to 0.12]	[0.04 to 0.23]
Controlling for GDP pc		X	X		X	X		X	X
Controlling for GHES pc and Pooled/THE			X			X			X
*N*	39	39	39	39	39	39	39	39	39

95% confidence intervals in brackets; *p < 0.05, ** *p* < 0.01, *** *p* < 0.001

Notes: *GDP pc* gross domestic product per capita, *GHES pc* government health expenditure as source per capita, *Pooled/THE* government and prepaid private spending as a share of total health expenditure. GDP pc and GHES pc are average over 2002–2004, reported in 2017 purchasing power parity international dollars. None of the three controls are statistically significant in any regression at the 0.05 level

**Fig. 3:**
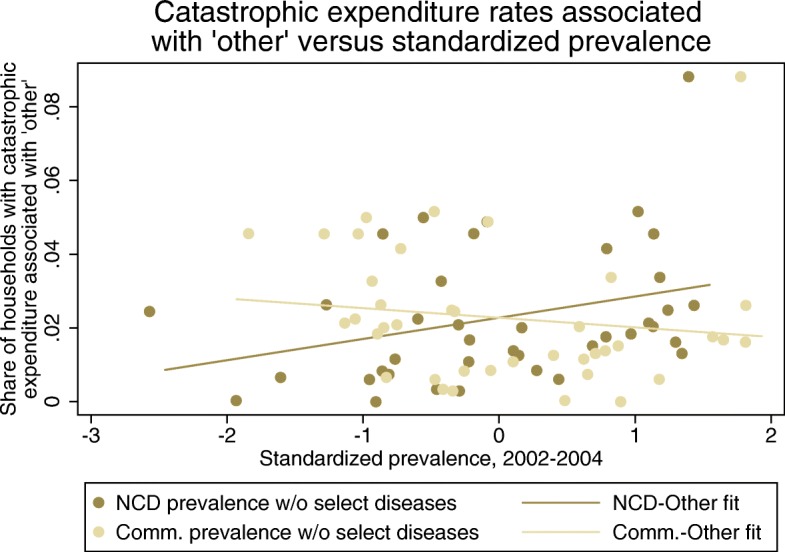
Catastrophic health expenditure (CHE) rates associated with “other” versus standardized prevalence. Catastrophic health expenditure rates in 39 countries among households with a respondent that used health care in the last 30 days in the World Health Survey. Noncommunicable disease (NCD) prevalence without selected diseases and communicable prevalence without selected diseases capture disease prevalence omitting the NCDs and communicable causes captured in the other disease-specific catastrophic health expenditure estimates. The full list of causes included in each category can be found in Additional file 1

Across income groups, a considerable fraction of households experienced CHE associated with a disease category not provided as a response option (“other”). This fraction increased with rising income. We could not unpack these causes based on the WHS data. However, in an effort to understand which areas of disease burden could be most associated with this category, we examined the linear association between other CHE and standardized and adjusted NCD and communicable disease prevalence. Other CHE declined as adjusted communicable prevalence increased (correlation of − 0.15; Fig. 3). In contrast, other CHE rose with adjusted NCD prevalence (correlation of 0.33). This suggests that the other category could include a substantial amount of NCD-associated CHE.

## Discussion

Our study characterized the distribution of CHE across disease areas in a comparable, comprehensive, and empirically based manner. The CHE distribution varied substantially across income levels: CHE cases associated with communicable diseases and maternal and child health declined with income whereas the portion of CHE cases attributed to heart disease and the “other” category rose with income. In countries with more pooling and higher income, fever, cough, diarrhea, and MCH comprised a smaller share of CHE, suggesting that these countries might do a better job at protecting people from these conditions but was also connected to lower prevalence rates of these conditions. In countries with more pooling and higher income, the CHE associated with heart disease was a higher share of total CHE. This is consistent with the higher prevalence of heart disease in countries with higher GDP per capita [27]. Furthermore, because heart disease can entail more intensive and expensive treatments, this finding is consistent with existing literature that emphasizes the role of availability of care, expensive technology and prices as potential drivers of higher CHE as income increases [5, 28]

The distribution of CHE by disease was much less distinct when comparing the poor to the non-poor. The uncertainty intervals for the CHE estimates associated with heart disease, injuries, and asthma overlapped, and the other category was also in a similar range for two population groups. This suggests that the epidemiologic profile of a country and context-specific health system features (e.g., costs, availability, and access to specific health services) may be more substantial drivers of disease-specific CHE than poverty status alone.

Across the disease areas that could be examined in depth, the CHE associated with heart disease exhibited the strongest relationship with share of the population afflicted. However, this relationship varied widely across countries, with a much larger spread (standard deviation) of CHE for CVD than for other areas—countries with similar rates of heart disease exhibited widely different proportions of the population affected by heart disease CHE. Furthermore, based on the regression results, for each percentage point increase in CVD prevalence, substantially more households incurred CHE than the other disease areas. The incidence of CHE associated with injuries and the crude birth rate was much lower. This suggests that where injuries and births affected a larger share of the population, health systems have developed financial risk protection measures for these causes. Relative to maternal causes and injuries, countries with a high burden of heart disease were not doing as well at preventing heart disease CHE. Countries’ financial risk protection measures have potentially not kept pace with heart disease prevalence, either by reducing the costs of heart disease treatment or providing insurance covering the costs of heart disease care.

The regression results suggest that the stronger relationship between population afflicted and heart disease CHE is not explained by basic determinants of health system performance: GDP per capita, GHES per capita, or the share of total health expenditure that is pooled—the slope of the relationship is unchanged when including these controls. This further emphasizes the role of price, availability of care, and technology as potentially driving up heart disease CHE. The costs for heart disease care tend to be more expensive on average than for other causes, and as care becomes available, insurance programs and other pooling mechanisms may not yet sufficiently cover these costs. The relationship between maternal CHE and the crude birth rate, in contrast, becomes stronger when controlling for GHES and pooled spending as a share of THE, indicating that some of the variation in maternal CHE is explained by how much is invested by the government and otherwise pooled to improve financial risk protection.

Another key finding was the substantial share of catastrophic spending associated with the “other” cause category. Across all WHS countries, an average of 45% of CHE cases could not be associated with a specific disease area. In the WHS, the options provided to respondents did not capture a range of diseases and conditions, including HIV/AIDS, malaria, diabetes, cancer, pneumonia, neglected tropical diseases, and general well visits. Respondents seeking care for these reasons would have to select the other response option. It is difficult to determine the distribution of these conditions in the other category. Plotting the CHE rates against disease burden suggested that much of this spending could be associated with NCDs, but more research is required in this area.

Data challenges comprised the main limitations of our study. First, the age of the WHS was a limitation—the survey was implemented over 2002–2004. Since this time, NCDs, including cardiovascular disease, have risen as a share of disease burden in low- and middle-income countries [27]. Ceteris paribus, this would increase the number of CHE cases associated with heart disease. However, a number of countries have implemented reforms to improve financial risk protection since this time, including expanding insurance schemes and eliminating user fees. While some of these efforts have affected all types of disease areas, a portion of schemes—particularly those funded by development assistance—have focused on maternal and child health and infectious diseases such as HIV/AIDS, tuberculosis, and malaria. For these reasons, it seems likely that the CHE associated with NCDs could have risen since that time. Second, respondents only reported the reason for using care at their most recent health care visit. A core assumption was that *all* household health spending in the last month was related to the health care associated with that visit. It is also likely that spending related to other diseases and conditions as well as for other family members was captured. Well-visit spending would typically be associated with the other category but could be included in household health expenditure as well. By honing in on health care in the last month only, we strengthened the validity of this assumption. Because we used visits in the last month only, we believe it was valid to assume the bulk of spending on health related to the disease or condition specified.

Other data limitations related to expenditure. First, the spending captured did not measure non-health spending related to illness onset, including transportation costs and opportunity costs (e.g., lost wages) associated with health care use. Second, the WHS captured a limited amount of household expenditure items and did not capture home production, resulting in underestimates of household expenditure. Third, the WHS was conducted over 2002–2004 and disease burden has changed substantially since [19]. Even so, the relationship between disease burden and CHE reflect core patterns that likely remain relevant today. Finally, comparing CHE across countries can be sensitive to important specifications such as the equivalency scale used to adjust for households, purchasing power parity adjustments, and the subsistence expenditure threshold [29].

Finally, our study was limited by the lack of more detailed information about the nature of health care delivery and the different health services available in the countries studied. Out-of-pocket spending on any of the disease areas studied is contingent on the availability of health services. If no heart disease treatment was available, afflicted individuals would not be able to spend on health care. Furthermore, we had no information about the severity of conditions and the quality and appropriateness of health care which mitigated symptoms or altered the course of disease.

## Conclusions

To achieve UHC, health systems will have to respond to challenges along multiple fronts, spanning the coverage, availability, and affordability of services. Countries may have prioritized financial protection in certain disease areas in the past because those disease areas were concentrated among the poor and of high priority on the international agenda. However, as populations develop diseases—such as heart disease, diabetes, and cancers—that the health system is not equipped to deliver in an affordable manner, households may increasingly face financial hardship. As countries pursue UHC, policymakers should pay attention to the newly emerging burden of NCDIs as a driver of CHE and consider comprehensive policies and benefits packages that provide financial risk protection across those disease areas.

## Additional file

Additional file 1: Supplementary web appendix. This file provides additional details on the data and methods employed in this analysis. (PDF 150 kb)

## References

[1] World Health Organization (WHO). Universal Health Coverage. Geneva: WHO. Available at: http://www.who.int/healthsystems/universal_health_coverage/en/. Accessed 7 Sept 2017

[2] United Nations. Sustainable Development Goal 3: Ensure healthy lives and promote well-being for all at all ages. Sustainable Development Goals Knowledge Platform. Available at: https://sustainabledevelopment.un.org/sdg3. Accessed 27 Jan 2019.

[3] Murray CJL, Frenk J (2000). A framework for assessing the performance of health systems. Bull WHO.

[4] Roberts M, Hsiao W, Berman P, Reich M (2008). Getting Health Reform Right: A Guide to Improving Performance and Equity.

[5] Wagstaff A, Flores G, Hsu J, Smitz MF, Chepynoga K, Buisman LR, van Wilgenburg K, Eozenou P (2017). Progress on catastrophic health spending in 133 countries: a retrospective observational study. Lancet Glob Health.

[6] Wagstaff A, Flores G, Smitz MF, Hsu J, Chepynoga K, Eozenou P (2018). Progress on impoverishing health spending in 122 countries: a retrospective observational study. Lancet Global Health.

[7] Saksena P, Hsu J, Evans DB (2014). Financial risk protection and universal health coverage: evidence and measurement challenges. PLoS Med.

[8] WHO and the World Bank (2015). Tracking universal health coverage: first global monitoring report.

[9] Bukhman G, Mocumbi AO, Horton R (2015). Reframing NCDs and injuries for the poorest billion: a lancet commission. Lancet.

[10] Institute for Health Metrics and Evaluation (IHME) (2017). Financing Global Health 2016: development assistance, public and private health spending for the pursuit of universal health coverage.

[11] Kankeu HT, Saksena P, Xu K, Evans DB (2013). The financial burden from non-communicable diseases in low- and middle-income countries: a literature review. Health Research Policy and Systems.

[12] WHO. Global Health Expenditure Database: National Reports. Geneva: WHO. Available at; http://apps.who.int/nha/database/DocumentationCentre/Index/en. Accessed 9 Oct 2017

[13] WHO. Global Health Expenditure Database: Documentation Centre. Geneva: WHO. Available at: http://apps.who.int/nha/database/DocumentationCentre/Index/en. Accessed 7 Sept 2017

[14] Essue B, Laba T-L, Knaul F, Chu A, Minh HV, Jamison DT, Gelband H, Horton S, Jha P, Laxminarayan R, Mock CN, Nugent R (2018). Economic Burden of Chronic Ill-Health and Injuries for Households in Low- and Middle-Income Countries. Disease Control Priorities (third edition). Volume 9, Disease Control Priorities: Improving Health and Reducing Poverty.

[15] Jan S, Laba TL, Essue BM, Gheorghe A, Muhunthan J, Engelgau M, Mahal A, Griffiths U, McIntyre D, Meng Q, Nugent R, Atun R (2018). Action to address the household economic burden of non-communicable diseases. Lancet.

[16] Verguet S, Memirie ST, OF N (2016). Assessing the burden of medical impoverishment by cause: a systematic breakdown by disease in Ethiopia. BMC Med.

[17] Engelgau MM, Karan A, Mahal A (2012). The economic impact of non-communicable diseases on households in India. Glob Health.

[18] Kankeu HT, Saksena P, Xu K, Evans DB (2013). The financial burden from non-communicable diseases in low- and middle-income countries: a literature review. Health Res Policy Syst..

[19] Xu K, et al. 2007.

[20] Alkire R*,* Santos S*.* 2011. Multidimensional poverty index 2011: brief methodological note*.* Oxford Poverty & Human Development Initiative (OPHI) Available at: http://www.ophi.org.uk/wp-content/uploads/MPI_2011_Methodology_Note_4-11-2011_1500.pdf?cda6c1. Accessed 9 June 2017

[21] GBD 2016 Disease and Injury Incidence and Prevalence Collaborators (2017). Global, regional, and national incidence, prevalence, and years lived with disability for 328 diseases and injuries for 195 countries, 1990–2016: a systematic analysis for the Global Burden of Disease Study 2016. Lancet.

[22] United Nations, Department of Economic and Social Affairs, Population Division (2017). World population prospects: the 2017 revision, DVD Edition.

[23] Musgrove P (1996). Public and private roles in health: theory and financing patterns. Health, Nutrition and Population (HNP) Discussion Paper.

[24] World Health Organization (WHO). Global Health Expenditure Database. Geneva: WHO. Available at: http://apps.who.int/nha/database. Accessed 25 Nov 2018

[25] Dieleman JL, Haakenstad A, Micah A, Moses M (2018). Spending on health and HIV/AIDS: Domestic health spending and development assistance in 188 countries, 1995–2015. Lancet.

[26] Raban MZ, Dandona R, Dandona L (2013). Variations in catastrophic health expenditure estimates from household surveys in India. Bull World Health Org.

[27] GBD Compare: Institute for Health Metrics and Evaluation (IHME) (2017). GBD Compare.

[28] Xu K, Evans DB, Carrin G, Aguilar-Rivera AM, Musgrove P, Evans T (2007). Protecting households from catastrophic health spending. Health Aff.

[29] Koch SF (2018). Catastrophic health payments: does the equivalence scale matter?. Health Policy Plann.

