# Supplementary material for: Disaggregating catastrophic health expenditure by disease area: cross-country estimates based on the World Health Surveys
Source: BMC Med. 2019 Feb 13;17:36. doi: 10.1186/s12916-019-1266-0 (PMC6372998; doi:10.1186/s12916-019-1266-0)
Supplement: Supplementary file 1 — Supplementary web appendix. This file provides additional details on the data and methods employed in this analysis. (PDF 150 kb) [file 12916_2019_1266_MOESM1_ESM.pdf]

Supplementary Web Appendix

1. Countries included in the analysis

Bangladesh, Bosnia and Herzegovina, Brazil, Burkina Faso, Chad, China, Cote d'Ivoire, Comoros, Congo, Dominican Republic, Ecuador, Georgia, Ghana, Guatemala, India, Kazakhstan, Kenya, Laos, Mali, Malawi, Malaysia, Mauritania, Mauritius, Mexico, Myanmar, Namibia, Nepal, Pakistan, Paraguay, Philippines, Senegal, South Africa, Sri Lanka, Swaziland, Tunisia, Ukraine, Vietnam, Zambia, Zimbabwe.

2. Methods for excluding disease cause categories

To calculate our adjusted measures of disease burden and compare to "other" CHE, we excluded the disease areas already included in the disease-specific catastrophic health expenditure estimates. The prevalence estimates could not be simply subtracted from the total NCD or communicable disease prevalence numbers because of the presence of co-morbidities across causes in the Global Burden of Disease study. We assumed independence across causes, which is consistent with the assumptions of independence across comorbidity simulations used in the GBD study, and then computed a total for the respective disease areas, represented in the following:

$$Total\ prevalence = 1 - \prod (1 - disease\ prevalence)$$

Where "Total prevalence" is prevalence (in percent terms) for communicable or noncommunicable diseases, respectively.

The modified version of communicable disease prevalence was represented by: Encephalitis, Food-borne trematodiases, Leishmaniasis, Leprosy, Lymphatic filariasis, Intestinal nematode infections, Onchocerciasis, Other neglected tropical diseases, Other communicable, maternal, neonatal, and nutritional diseases, Schistosomiasis, and Trachoma.

For NCDs, modified prevalence was captured by: Cirrhosis and other chronic liver diseases, Diabetes, urogenital, blood, and endocrine diseases, Digestive diseases, Gout, Low back and neck pain, Mental and substance use disorders, Neoplasms, Neurological disorders, Other musculoskeletal disorders, Congenital birth defects, Skin and subcutaneous diseases and Sense organ diseases.

3. Computing the poverty index (PI)

We used a modified version of the multidimensional poverty index (MPI) to compute our poverty index (PI), which was used to determine whether a household was poor.[1] Because our study focused on health, we omitted the health components of the MPI – nutrition and mortality. Because the World Health Survey did not capture whether household members below the age of 12 were attending school, we were also unable to assess school attendance, and thus relied only on the years of schooling reported for adults as the education component of the index. A household was considered poor if it ws deprived in four or more of the indicators. Table A1 reports the definitions of the indicators used to assess deprivation.

Table S1: Indicators used in the poverty index (PI)

**Indicators**	**Definition**
**Education**	
**Years of schooling**	No household member aged ≥ 10 years has completed ≥ 5 years of schooling.
**Living standards**	
**Cooking fuel**	The household cooks with dung, wood or charcoal.
**Improved sanitation**	The household does not have a flush toilet or latrine, or does not have or must share one of the following with other households: a ventilated improved pit or composting toilet.
**Safe drinking water**	The household does not have piped water, a public tap, a borehole or pump, a protected well or spring or rainwater within a 30 minutes roundtrip walk.
**Electricity**	The household has no electricity.
**Floor**	The household has a dirt, sand or dung floor.
**Assets**	The household does not own more than one of: radio, TV, telephone, bike, motorbike or refrigerator and does not own a car or truck.

Source: Alkire and Santos. 2011.

Table S2: Bootstrapped mean, 5th and 95th percentiles for the cases of catastrophic health expenditure by disease category and income group.

	**Fever, diarrhea and cough**	**Other maternal and child health**	**Heart disease**	**Injuries**	**Surgery**	**Asthma**	**Other**
LICs	49.2%	3.1%	2.3%	5.0%	0.8%	2.9%	36.7%
	(UI: 46.4-52.3%)	(UI: 2.5-3.8%)	(UI: 1.4-3.2%)	(UI: 4.0-6.2%)	(UI: 0.5-1.1%)	(UI: 2.0-3.9%)	(33.7% - 39.6%)
LMICs	28.1%	4.4%	5.2%	5.4%	1.2%	3.2%	52.4%
	(UI: 24.4-32.0%)	(UI: 2.8-6.2%)	(UI: 4.0-6.6%)	(UI: 3.6-7.7%)	(UI: 0.5-2.0%)	(UI: 1.9-4.7%)	(UI: 48.3-56.5%)
UMICs	23.7%	6.3%	10.1%	4.5%	1.2%	2.4%	51.8%
	(UI: 21.7-25.8%)	(UI: 4.6-8.0%)	(UI: 7.9-12.3%)	(UI: 3.4-6.0%)	(UI: 0.8-1.7%)	(UI: 1.2-3.8%)	(UI: 49.0-54.7%)
All	37.6%	3.9%	4.1%	5.2%	1.0%	3.0%	45.1%
	(UI: 35.4-39.9%)	(UI: 3.0-4.9%)	(UI: 3.3-4.9%)	(UI: 4.2-6.4%)	(UI: 0.6-1.4%)	(UI: 2.2-3.9%)	(UI: 42.6-47.6%)

Notes: LICs: Low-income countries; LMICs: Lower-middle-income countries; UMICs: Upper-middle-income countries; according to 2014 World Bank income classifications. UI: uncertainty interval based on 1000 bootstrapped draws.

Table S3: Bootstrapped mean, 5th and 95th percentiles for the cases of catastrophic health expenditure by disease category and poverty status.

	**Fever, diarrhea and cough**	**Other maternal and child health**	**Heart disease**	**Injuries**	**Surgery**	**Asthma**	**Other**
Non-Poor	35.8%	4.6%	4.2%	4.7%	1.0%	3.2%	46.4%
	(UI: 33.1-38.8%)	(UI: 3.5-5.8%)	(UI: 3.3-5.3%)	(UI: 3.6-6.0%)	(UI: 0.6-1.5%)	(UI: 2.2-4.4%)	(UI: 43.5-49.0%)
Poor	42.3%	1.5%	3.8%	4.8%	0.4%	8.3%	38.8%
	(UI: 34.6-50.4%)	(UI: 1.1-2.1%)	(UI: 0.8-5.8%)	(UI: 2.8-7.9%)	(UI: 0.1-0.7%)	(UI: 3.0-14.8%)	(UI: 31.1- 47.5%)

Notes: UI: uncertainty interval based on 1000 bootstrapped draws.

References

1. Alkire R, Santos S. 2011. Multidimensional Poverty Index 2011: Brief Methodological Note. Oxford Poverty & Human Development Initiative (OPHI). Available at: http://www.ophi.org.uk/wp-content/uploads/MPI_2011_Methodology_Note_4-11-2011_1500.pdf?cda6c1 (Accessed June 9, 2017).
